# Characterization of MtoD from *Sideroxydans lithotrophicus:* a cytochrome c electron shuttle used in lithoautotrophic growth

**DOI:** 10.3389/fmicb.2015.00332

**Published:** 2015-04-28

**Authors:** Christopher R. Beckwith, Marcus J. Edwards, Matthew Lawes, Liang Shi, Julea N. Butt, David J. Richardson, Thomas A. Clarke

**Affiliations:** ^1^Centre for Molecular and Structural Biochemistry, School of Biological Sciences and School of Chemistry, University of East AngliaNorwich, UK; ^2^Pacific Northwest National LaboratoryRichland, WA, USA

**Keywords:** class 1 cytochrome, MtoD, *Sideroxydans lithotrophicus* ES-1, iron oxidation

## Abstract

The autotrophic *Sideroxydans lithotrophicus* ES-1 can grow by coupling the oxidation of ferrous iron to the reduction of oxygen. Soluble ferrous iron is oxidized at the surface of the cell by an MtoAB porin-cytochrome complex that functions as an electron conduit through the outer membrane. Electrons are then transported to the cytoplasmic membrane where they are used to generate proton motive force (PMF) (for ATP synthesis) and NADH for autotrophic processes such as carbon fixation. As part of the *mtoAB* gene cluster, *S. lithotrophicus* also contains the gene *mtoD* that is proposed to encode a cytochrome c protein. We isolated *mtoD* from a *Shewanella oneidensis* expression system where the *mtoD* gene was expressed on a pBAD plasmid vector. Biochemical, biophysical, and crystallographic characterization of the purified MtoD revealed it as an 11 kDa monomeric protein containing a single heme. Sequence and structural alignment indicated that MtoD belonged to the class-1 cytochrome c family and had a similar fold to ferricytochrome c552 family, however the MtoD heme is bis-histidine coordinated and is substantially more exposed than the hemes of other family members. The reduction potential of the MtoD heme at pH 7 was +155 mV vs. Standard Hydrogen Electrode, which is approximately 100 mV lower than that of mitochondrial cytochrome *c*. Consideration of the properties of MtoD in the context of the potential respiratory partners identified from the genome suggests that MtoD could associate to multiple electron transfer partners as the primary periplasmic electron shuttle.

## Introduction

The potential for bacteria to utilize iron as an energy source has been widely recognized in recent years (Bird et al., 2011; Konhauser et al., 2011). Several families of bacteria live at the microoxic/ferrous iron interface where they can survive by coupling the oxidation of ferrous iron to the reduction of oxygen (Hedrich et al., 2011). These bacteria are typically autotrophic and generate sufficient energy from this reaction to fix carbon dioxide and dinitrogen. Bacteria that are capable of this chemolithoautotrophic growth include acidophilic bacteria involved in acid mine drainage such as *Acidothiobacillus ferrooxidans* and *Leptospirillum ferrooxidans* (Rawlings et al., 1999; Ferguson and Ingledew, 2008; Mishra and Rhee, 2014); the marine stalk-forming *Gallionella ferruginea* and the freshwater *Gallionella capsiferriformans* ES-2 and *Sideroxydans lithotrophicus* ES-1 (Hallbeck et al., 1993; Emerson and Moyer, 1997). The genomes of these organisms have been sequenced revealing a range of putative metabolic pathways (Emerson et al., 2013) and analysis of these pathways poses a range of interesting questions: how do the bacteria extract the electrons from the ferrous iron, and how are those electrons ultimately coupled to the generation of NADH and a proton motive force (PMF)?

The best studied of the iron oxidizing bacteria is the acidophilic *A. ferrooxidans*, where an outer membrane monoheme Cyc2 collects electrons from the oxidation of Fe(II) to Fe(III) and transfers them to a periplasmic rusticyanin which then transfers the electrons to two potential shuttles, the diheme cytochromes Cyc1 and CycA1. These diheme cytochromes then transfer the electrons down divergent routes, either to a cytochrome bc1 complex where electrons enter the quinol pool to ultimately generate NADH, or an aa_3_ oxidase where oxygen is reduced to water together with the transport of protons across the membrane (Bonnefoy and Holmes, 2012; Roger et al., 2012).

The neutrophilic *S. lithotrophicus* ES-1 does not contain any genes with significant homology for *cyc2*, but instead contains the genes *mtoA (Slit_2497)* and *mtoB (Slit_2496)* (Liu et al., 2012). These are homologs of *Shewanella oneidensis mtrA* and *mtrB*, which encode a decaheme cytochrome MtrA and transmembrane porin MtrB. These two proteins form a porin-cytochrome complex in the outer membrane of *S. oneidensis* that allows for efficient electron transport through the outer membrane via a chain of hemes (Hartshorne et al., 2009; White et al., 2013). It has been proposed that MtoA and MtoB fulfill a similar function in *S. lithotrophicus* ES-1, in forming an electron conduit that allows electrons to be collected from the oxidation of ferrous iron at the surface of the cell and transported through to the periplasm (Liu et al., 2012).

Both *mtoA* and *mtoB* are located in a gene cluster in the *S. lithotrophicus* ES-1 genome that also contains two other *c-*type cytochromes; *Slit_2498* that encodes MtoD, a small mono-heme cytochrome and *Slit_2495*, which encodes CymA_ES−1_, a tetraheme quinol oxidoreductase. As part of the same cluster it is possible that all four of these genes are co-transcribed and expressed as part of an operon. This would provide a porin-cytochrome complex (MtoAB), a soluble periplasmic cytochrome (MtoD), and a quinol oxidoreductase in the cytoplasmic membrane (CymA_ES−1_) (Emerson et al., 2013).

*S. lithotrophicus* ES-1 also contains the genes necessary to express two distinct oxygenases, a cytochrome bb_3_ oxidase (cbb_3_), and a cytochrome bd_1_ oxidase (cbd_1_). These have been characterized in other bacteria and shown to have low *K_M_*-values for oxygen: typically cbd_1_ operates in the micromolar range while cbb_3_ operates in the sub-micromolar range. cbb_3_ couples O_2_ reduction to proton pumping and receives electrons from a cytochrome c, while cbd_1_ has no proton pumping mechanism and receives electrons directly from the quinol pool (Pitcher and Watmough, 2004).

The bioenergetic and biochemical mechanism of precisely how iron oxidizing bacteria are able to couple Fe oxidation to O_2_ reduction is still unclear. How do electrons from the MtoAB complex enter the cytochrome oxidase? It was previously suggested that CymA_*ES*−1_ was the redox partner, however, this would result in the loss of protonmotive force. In order to better understand the possible roles of the periplasmic redox partners in *S. lithotrophicus* ES-1 the MtoD cytochrome was expressed in a recombinant form, purified and characterized using a range of biochemical, spectroscopic, and crystallographic techniques.

## Materials and methods

### Expression and isolation of strep-II tagged MtoD

The 354 bp sequence encoding *mtoD* was synthesized and cloned into a puc57 vector by GENScript. *mtoD* was amplified from pUC57 using the following primers: *mtoD*_1_F: 5′-ATG ACT CGT CAA GCT TAT TCC TCA ATG TTG and *mtoD*_1_R: 5′-GAG CGA AAG GAT CCA GTC CAC CAG. A second pair of primers were used to make modifications to *mtoD* including the addition of a 5′ CACC overhang, making the *mtoD* insert compatible with the desired pBAD202 D-TOPO cloning kit, followed by a ribosome binding sequence and a 3′ 6xCAC repeat coding for a C-terminal polyhistidine tag. *mtoD*_2_F: 5′-CAC CTA AGA AGG AGA TAT ACA TCC CAT GAC TCG TCA AGC TTA TTC. *mtoD*_R_6xHis: 5′-CTA GTG GTG GTG GTG GTG GTG GAG CGA AAG GAT C. A pBAD directional TOPO® expression kit was used clone *mtoD*-His into a pBAD202 expression vector. One Shot TOP10 *E. coli* cells were transformed with pBAD202_*mtoD-*His using methods described in the pBAD D-TOPO user guide. pBAD202_*mtoD*-His was conjugated from TOP10 to *S. oneidensis* MR-1 using *E. coli* helper strain DH5α pRK2013. Kanamycin and carbenicillin were used to select for successfully conjugated MR-1. DNA sequencing performed by Eurofins MWG operon using primers *mtoD*_2_F and *mtoD*_R_6xHis confirmed successful conjugation of pBAD202_*mtoD*-His into *S. oneidensis* MR-1. For the production of Strep II-tagged MtoD pBAD202_*mtoD*-His was isolated from *S. oneidensis* MR-1 using a miniprep kit and the whole plasmid was amplified using the primers *mtoD*_*SII*_F: 5′-AAT TCG AGA AGT AGA AGG GCG AGC TCA AGC TTG AAG GTA and *mtoD*_SII_R: 5′-GTG GAT GAG ACC AGA GCG AAA GGA TCC AGT CCA CAG G. The pBAD202_*mtoD-*His template was removed by DpnI digestion followed by PCR clean up. T4 polynucleotide kinase was used to phosphorylate the linear product and a blunt end ligation was performed using DNA ligase to circularize the pBAD202_*mtoD*-*SII* product. One Shot TOP10 *E. coli* were transformed with pBAD202_*mtoD*-*SII* and conjugation was used to produce recombinant *S. oneidensis* MR-1 as before.

Recombinant *S. oneidensis* MR-1_*mtoD*-*SII* was cultured aerobically at 30°C in 20 L batches in LB media. Expression of tagged protein was induced at mid-exponential phase (OD_600_: 0.5) by addition of L-arabinose to a working concentration of 2 mM. Cultures were incubated for a further 5 h and harvested by centrifugation at 6000 *g* for 15 min. Recombinant MR-1 cell pellets were re-suspended in 20 mM HEPES pH 7 buffer and three passes of French Press treatment at 1000 psi (6.89 MPa) were used to lyse the cells. The soluble cell fraction was isolated by ultracentrifugation of the lysate at 205,000 *g* for 2 h. The supernatant was retained for purification of tagged MtoD. MtoD was isolated using 5 mL Strep-Tactin affinity column (GE healthcare). The column was run with a 20 mM HEPES pH 7, 150 mM NaCl equilibration/wash buffer. Four cycles of loading and eluting were performed to isolate all of the expressed MtoD. SDS PAGE was used to analyze eluted fractions; MtoD fractions were dialysed with 20 mM HEPES pH 7, 150 mM NaCl overnight then concentrated in preparation for size exclusion chromatography. Gel filtration was performed using a Superdex S75 16/60 column. The column was equilibrated with 20 mM HEPES pH 7, 150 mM NaCl before loading MtoD and running at a flow rate of 0.5 mL.min^−1^. Pure fractions of MtoD, determined by SDS PAGE analysis, were pooled, concentrated and dialysed with 20 mM HEPES pH 7, 100 mM NaCl overnight at 4°C.

### Pyridine hemochrome analysis of MtoD

Oxidized spectra of horse heart cytochrome c and MtoD were prepared in 20 mM HEPES pH 7 buffer containing 2 mM CaCl_2_ for cytochrome c, and 100 mM NaCl for MtoD. Each sample was fully oxidized with 10 μM K_3_Fe(CN)_6_ before measurement of UV/vis electronic absorption spectrum. The pyridine hemochrome method was used to quantify the concentration of heme in the purified MtoD sample and to determine the extinction coefficient of the Soret maxima of MtoD (Berry and Trumpower, 1987). Briefly cytochrome c and MtoD were mixed with pyridine and NaOH to give final concentrations of 3 and 75 mM, respectively. These samples were divided into two Suba-sealed quartz cuvettes. Samples were oxidized using 10 μM K_3_Fe(CN)_6_ and reduced with 3 mM Na_2_S_2_O_4_ to give the oxidized and reduced spectra of heme bis-pyridine. A Bio-Rad protein assay kit was used to determine the total protein concentration.

### Sedimentation velocity

Four hundred and ten microliters of MtoD samples of 3.5 and 8.5 μM were measured into sample chambers of a two chamber cell assembly and 20 mM HEPES pH 7, 100 mM NaCl was measured into the reference chambers. A Beckman Optima XL-A analytical ultracentrifuge with an An50Ti rotor was used to optically measure sedimentation, monitoring absorbance at 406 nm with a rotational speed of 42,000 rpm. 300 radial scans were performed over a 20-h period at a constant temperature of 20°C. Analyses of the sedimentation velocity data were performed using SEDFIT (Brown and Schuck, 2006). The C(s) distribution model was applied to the data and non-linear fitting was performed. Buffer density and viscosity parameters (1.0039 g.mL^−1^ and 1.0264 × 10 ^−2^ ρ, respectively) in addition to predicted v-bar for MtoD (0.7282 mL.g^−1^), were included in the C(s) model to solve Lamm equations.

### MtoD crystallization and data collection

A pure solution of MtoD was concentrated to 30 mg.mL^−1^ and sparse matrix screening, using the sitting drop method, was performed to explore potential crystallization conditions. Crystals formed at 16°C in 0.6 μl drops at ratios of 1:1 or 2:1 mother liquor/protein. The mother liquor was 0.1 M KCN, 30% PEG 2000 MME. Crystals were cryo-protected by transferring to a solution of mother liquor containing 12% glycerol before being vitrified by plunging into liquid nitrogen. Data were collected on MtoD crystals in a gaseous stream of nitrogen at 100 K on beamlines I03 at the Diamond Light Source (UK). MtoD crystals were of space group P22_1_2_1_ with typical cell dimensions of *a* = 29.70, *b* = 40.20, *c* = 92.180 Å. A SAD dataset was collected at a wavelength of 1.72 Å to a final resolution of 2.5 Å. Further datasets from single crystals were collected using an x-ray wavelength of 0.97 Å.

### MtoD structure determination and refinement

MtoD datasets were processed using XIA2 (Winter, 2010). The SAD dataset of MtoD was analyzed using the autosol pipeline within the PHENIX software suite (Adams et al., 2010). The program HySS located one heavy atom site and the electron density maps calculated with PHASER/RESOLVE were sufficiently interpretable to manually place a single heme corresponding to a single MtoD molecule in the asymmetric unit. The model building program Phenix AutoBuild was used to build residues followed by alternating rounds of manual building and refinement using PHENIX (Adams et al., 2010) or REFMAC (Murshudov et al., 2011). The final model was refined to an Rcryst (Rfree) value of 19.4 (24.5)%. This model has no residues in the disallowed region of the Ramachadran plot. Coordinates have been deposited in the RCSB Protein Data bank under accession code 4XXL.

### Electrochemistry

Mediated redox spectropotentiometry of a solution of MtoD was performed using methods described previously (Bamford et al., 2002). Experiments were performed under continuous argon gas flow with a solution of 5.5 μM MtoD in 20 mM HEPES pH 7, 100 mM NaCl containing 10 μM each of the following mediators: 2,3,5,6-tetramethyl-*p*-phenylenediamine (DAD), phenazine methosulphate (PMS), phenazine ethosulphate (PES), 5-hydroxy-1,4-napthoquinone, 2,3,5,6-tetramethyl-1,4-benzoquinone, 2-methyl-1,4-napthoquinone, 9,10-anthraquinone-2,6-disulphonic acid, anthraquinone-2-sulphonic acid and 1,1′-dibenzyl-4,4′-bipyridinium dichloride. The electrochemical potential was raised or lowered through the addition of aliquots of anaerobic solutions containing potassium ferricyanide or sodium dithionite, respectively.

## Results

### Expression, purification, and characterization of S. lithotrophicus MtoD

**Figure 1:**
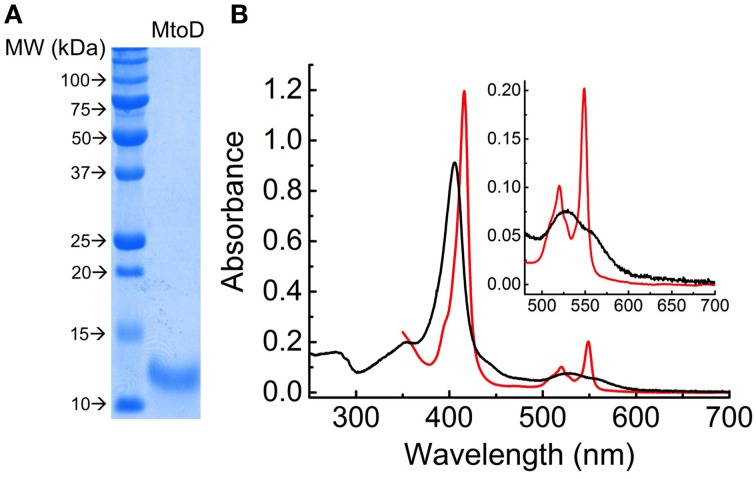
**Isolation and preliminary characterization of recombinant MtoD: (A) SDS-polyacrylamide gels of Coomassie stained MtoD**. **(B)** UV-visible wavelength spectrum of fully oxidized (black) and fully reduced (red) MtoD. MtoD was prepared by addition of potassium ferricyanide to obtain a fully oxidized spectrum and addition of sodium dithionite to obtain a fully reduced spectrum. ***Inset*** the region between 500 and 700 shows the αβ peaks associated with the c-type heme.

MtoD was purified to homogeneity from *S. oneidensis* as described in Methods. SDS-PAGE analysis revealed that MtoD ran as a single band with apparent molecular weight of 13 kDa, slightly larger than the predicted molecular weight of 11 kDa due to the c-terminal strep-II tag (Figure 1A). Edman degradation (PNAC facility, Cambridge UK) revealed the N-terminal sequence began AVDVD, matching the cleavage site predicted by SignalP (Petersen et al., 2011) and pyridine hemochrome assays revealed that the sample of MtoD contained approximately stoichiometric ratio of 0.85 heme: protein, giving an ε_410_ coefficient of 105.2 mM^−1^ cm^−1^.

**Figure 2:**
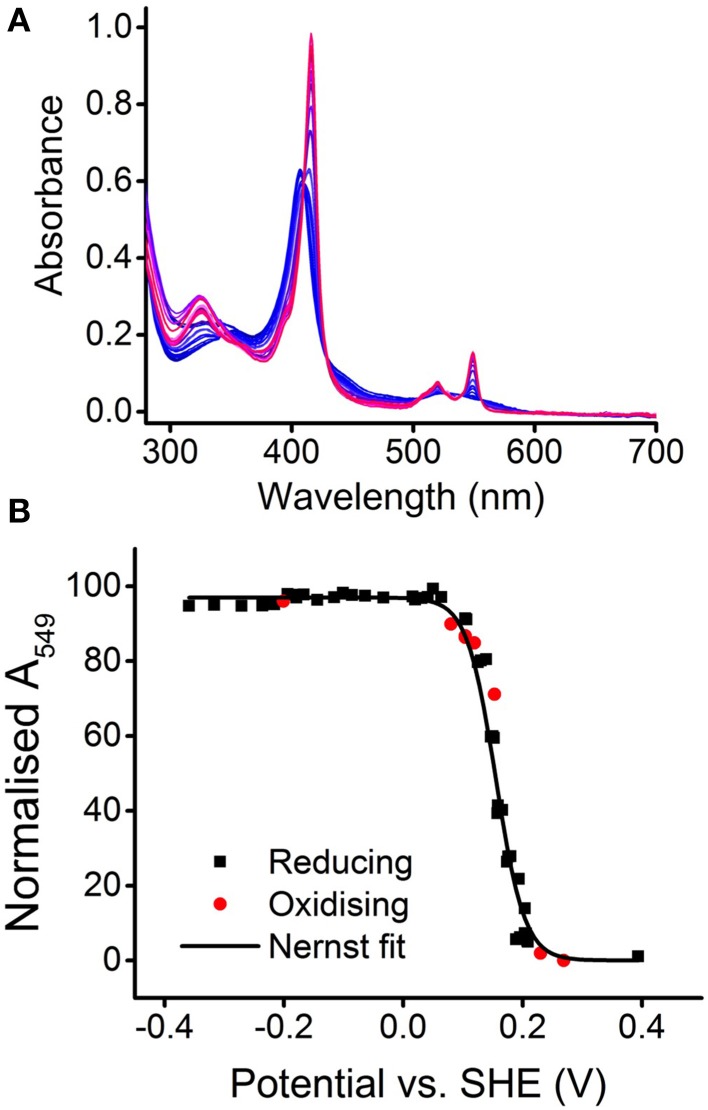
**Redox characterization of MtoD. (A)** Electronic absorbance of MtoD equilibrated at potentials between 0.4 and −0.4 V during a mediated potentiometric titration. Spectra are colored from blue to red corresponding to increasingly reduced protein. **(B)** Variation of absorbance at 549 nm (A_549_) with solution potential (points) and best fit (line) to the Nernst equation for a one-electron transformation with a mid-point potential of +155 mV vs. SHE. Experiments performed in 20 mM HEPES pH 7, 100 mM NaCl at 21°C with a mediator range as listed in the methods.

Solutions of MtoD display electronic absorbance spectra consistent with the presence of a low-spin c-type heme (Figure 1B). The characteristic Soret peak in the oxidized protein has a maximum at 406 nm, giving a 280/406 nm absorbance ratio of 0.19. The 406 nm maximum shifts to 416 nm on reduction of MtoD and α and β peaks appear with maxima at 549 and 520 nm, respectively. Spectral features above 600 nm would be indicative of high-spin ferric heme or low-spin ferric heme with His/Met axial heme ligation as is typically observed in other class-1 cytochrome-c family members (Bertini et al., 2006). However, MtoD displayed no detectable absorbance above 600 nm in agreement with the heme ligand set resolved by X-ray diffraction as described below. Mediated potentiometric titration of MtoD monitored by electronic absorbance spectroscopy defined the heme redox properties (Figure 2A). Changes in the absorbance at 549 nm due to the ferric/ferrous heme interconversion were fully reversible with change of solution potential (Figure 2B). The behavior was in excellent agreement with that predicted by the Nernst equation for a single redox center undergoing a one-electron redox transformation with a mid-point potential of 155 ± 10 mV vs. SHE.

**Figure 3:**
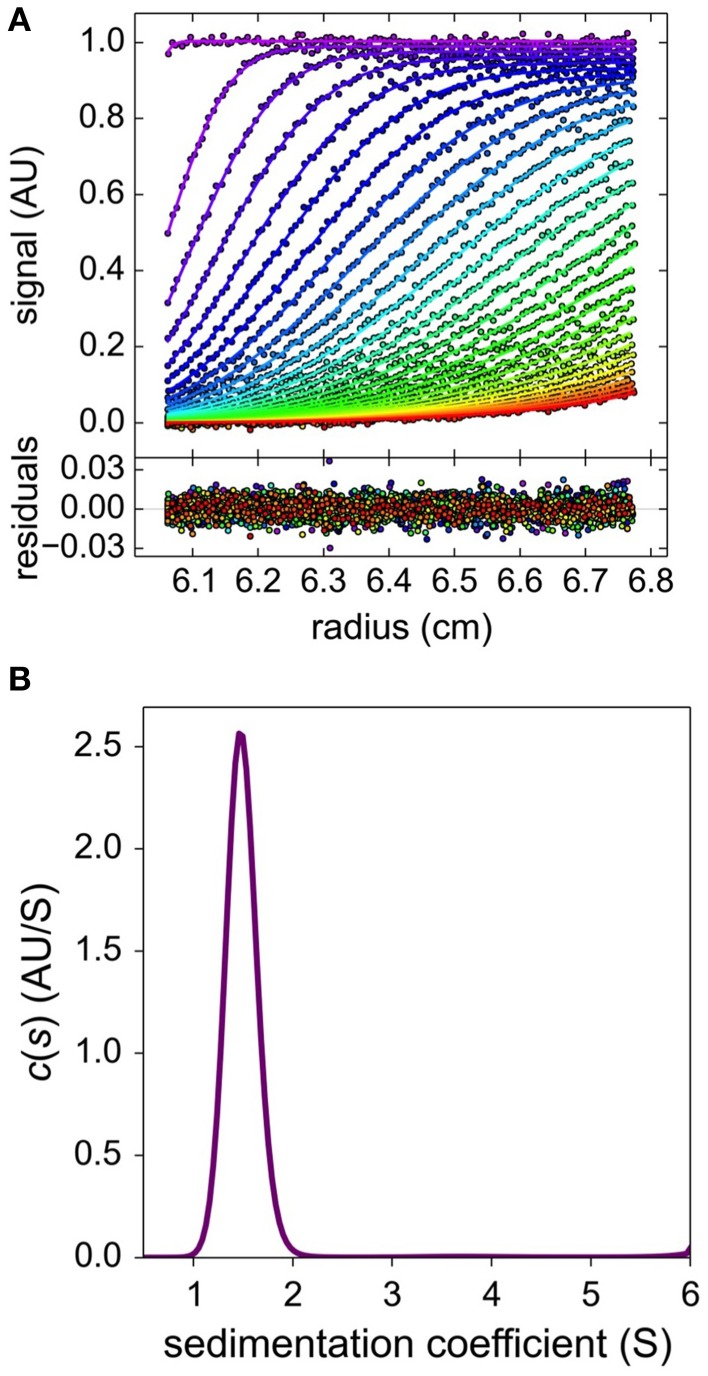
**Solution based biophysical characterization using sedimentation velocity. (A)** Absorption at 406 nm was used to track the boundary migration of an 8.5 μM sample of MtoD in 20 mM HEPES pH 7, 100 mM NaCl at 42,000 rpm. The change in absorbance was fitted using SEDFIT with the residual fit shown below the data **(B)**. The c(s) distribution of the fitted data indicates the presence of a single, non-interacting species with a sedimentation coefficient of 1.55 s and a corresponding molecular weight of 11.7 kDa, corrected for temperature and buffer parameters.

The biophysical properties of MtoD in solution were examined using sedimentation velocity. MtoD samples at 3.5 and 8.5 μM were centrifuged as described in methods and the migration profile of MtoD at 406 nm was measured over 5 h (Figure 3A). The data was fitted using the software program SEDFIT which revealed a single species with a sedimentation coefficient of 1.55 s and a molecular mass of 11.7 kDa (Figure 3B). Further analysis of the sedimentation data gave a f/f_*o*_ coefficient of 1.24, indicating that MtoD behaved like a monomeric globular protein in solution.

**Figure 4:**
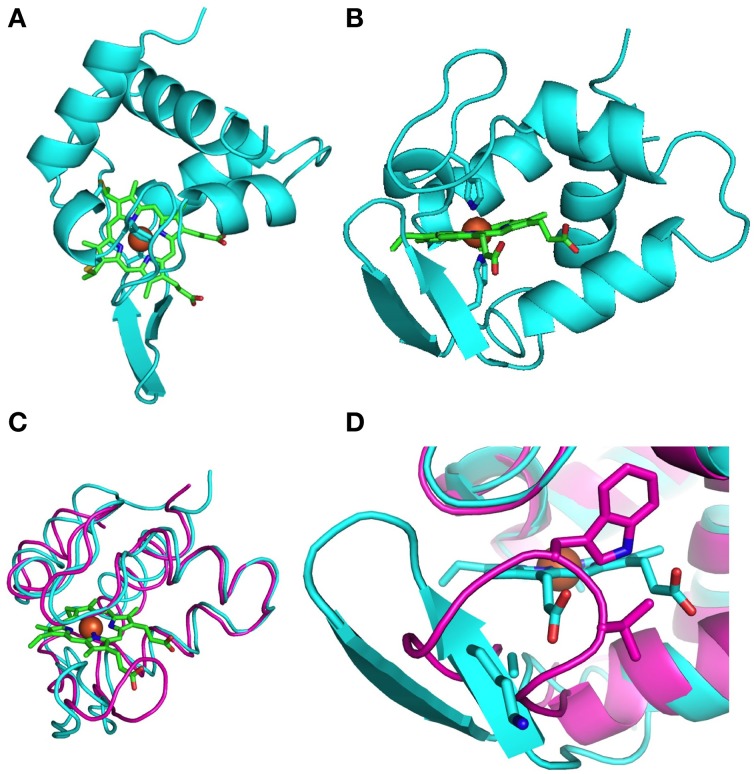
**1.47Å Crystal structure of MtoD. (A)** Top view of MtoD; **(B)** Side view of MtoD revealing the near-parallel histidine side chains co-ordinating the heme iron. **(C)** Superposition of MtoD (cyan) over the secondary structure of cytochrome c552 from *Hydrogenbacter thermophilus* (magenta) **(D)** Close up of the loop region that exposes the MtoD heme. In cytochrome c552 the conserved tryptophan Trp54 covers much of the exposed heme.

The crystal structure of MtoD was solved to an initial resolution of 2.29 Å by single wavelength anomalous dispersion (SAD) using the anomalous signal caused by the single iron atom contained within the heme group. The initial model was used as a template for molecular replacement to obtain a final resolution of 1.47 Å. The overall statistics obtained for data collection and structure refinement are given in Supplementary Table 1. The crystal structure contained residues 28–119 of the processed amino acid sequence and a single c-type heme covalently attached to Cys_43_ and Cys_46_ via thioether bonds (Figure 4A), consistent with the histidine ligands predicted from the sequence alignment. The iron atom of the heme group was coordinated by the imidazoles of the porin cofactor and His_47_ and His_95_ of the active site. The imidazole side chains of the two histidines coordinating the heme iron are arranged near-parallel relative to one another, at an angle of approximately 35° (Figure 4B).

The sequence alignment between MtoD and the other structurally resolved cytochromes revealed that cytochrome c552 from *Nitrosomas europea* had the highest amino acid sequence homology (Supplemental Figure 1). When the structure of MtoD was compared with the other available structures of bacterial monoheme c-type cytochromes, the closest structural homolog was cytochrome c552 from *Hydrogenobacter thermophilus* (PDB id. 1YNR). Superposition of the main chain of this cytochrome with MtoD gave an average root-mean-square-displacement of 2.59 Å (Figure 4B, Table 1). Despite the high level of secondary structure conservation there are notable structural differences between MtoD and the other known c-type cytochromes. One obvious difference is that the axial coordination of the MtoD heme is bis-Histidine, while other crystallized cytochromes have Histidine/Methionine coordination (Table 1). The class 1 cytochromes typically contain a flexible loop that covers the front of the heme, however in MtoD this region is restrained by a hydrogen bonding network that causes the formation of a β-loop and prevents the peptide sidechains from interacting with the heme propionate groups (Figures 4C,D). This causes an increase in the overall surface exposure of the MtoD heme, giving an overall exposed heme of 152 Å^2^ compared to 30–61 Å^2^ for other class 1 cytochromes. Typically the hemes of these class 1 cytochromes are exposed on one side, next to the thiolated cysteine residues, with the propionate edge being completely covered. To date the heme group of MtoD is significantly more exposed to solvent than the heme of any other structurally resolved monoheme cytochrome, suggesting that the properties of the heme are likely to be extremely susceptible to changes within the local environment.

**Table 1:** **Structural comparison of MtoD with similar monoheme c-type cytochromes**.

**Name**	**Class**	**PDB id**	**Axial ligands**	**Accesible Heme area (Å^2^)**	**RMSD (Å)**
*S. lithotrophicus* MtoD	–	4XXL	His/His	152.5	–
*N. europea* c552	c552	1A56	His/Met	54.2	3.23
*P. aeruginosa* c551	c551	451C	His/Met	52.6	3.25
*H. thermoluteolus* c552	c552	2D0S	His/Met	61.3	2.87
*H. thermophilus c552*	c552	1YNR	His/Met	57.4	2.59
*P. stutzeri c551*	c552	1COR	His/Met	55.2	3.08
*A. aeolicus c555*	c555	2ZXY	His/Met	37.5	3.54

*Six cytochromes were identified through sequence similarity to MtoD using BLAST. Structures were downloaded from the RCSB protein data bank. The root mean square displacement (RMSD) was measured using SUPERPOSE (Krissinel and Henrick, 2004). Accesible heme area was measured using a 1.4 Å probe using AREAIMOL (Shrake and Rupley, 1973)*.

## Discussion

Autotrophic iron oxidizing bacteria face a significant bioenergetic challenge in generating both the reducing equivalents (NADH) and chemical energy (ATP) required for carbon fixation and other anabolic reactions essential for cell survival. The energy source for both NADH and ATP production is generated from the liberation of electrons obtained from the oxidation of iron at the cell surface. At pH 7.0 the redox potential of this iron couple Fe(OH)_3_/Fe^2+^ is -236 mV vs. SHE, which is low enough to allow electron transfer across the outer membrane through the MtoAB complex (Widdel et al., 1993; Liu et al., 2012). The electrons obtained from iron oxidation are required both for PMF-coupled oxygen reduction, and the reduction of NADH at the cytoplasmic membrane. At an initial potential around −236 mV the energy associated with these electrons would be sufficient to catalyze the reduction of oxygen (+816 mV), but they would not be able to reduce NAD^+^ to NADH (−320 mV). As a consequence electrons destined for NADH formation will have to be pumped “uphill” through different carriers before reducing NADH. The energy source for this uphill electron transfer is most likely to be obtained from the proton gradient. In *A. ferrooxidans* two periplasmic diheme cytochromes Cyc1 and CycA1 take electrons to either the cytochrome oxidase or the cytochrome bc_1_ complex, which then runs in reverse to reduce ubiquinone. These cytochromes are located in two separate operons; the *rus* operon contains *cyc1* as well as *cyc2, rusA* and the genes for a cytochrome c oxidase, while the *petl* operon contains *cycA1* and the genes for a cytochrome bc_1_ complex (Roger et al., 2012). In contrast the *mto* gene cluster of *S. lithotrophicus* contains the gene for only one monoheme cytochrome: *mtoD*, while the gene clusters that contain the genes of cytochrome bc_1_ and cytochrome bb_3_ do not contain any soluble monoheme cytochromes.

**Figure 5:**
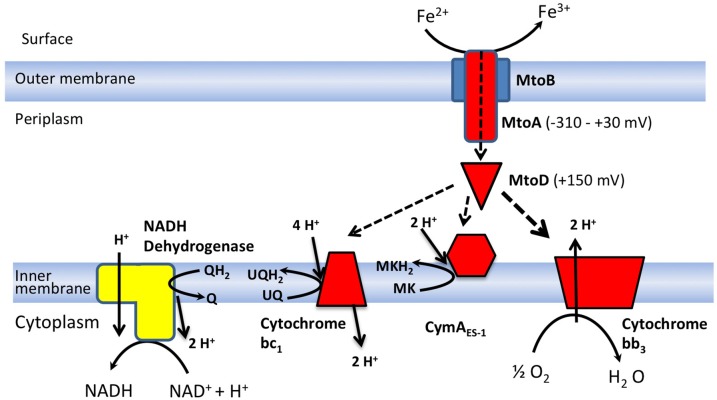
**Proposed dual role of MtoD in supporting both proton translocation and NADH production**. Possible electron transfer pathways through MtoA and MtoD are indicated by dashed lines. UQ and UQH_2_ refer to ubiquinone and ubiquinol respectively, while MK and MKH_2_ refer to menaquinone and menaquinol. Q and QH_2_ represent non-specific quinones and quinols. The known redox midpoint ponentials for *S. lithotrophicus* ES-1 MtoD and MtoA are shown in parentheses.

The MtoD cytochrome has a number of unusual features. Comparative analysis of the MtoD amino acid sequence with the sequences of other structurally defined class-1 cytochromes revealed that MtoD is unusual in having bis-His coordination instead of His/Met, and that the sequence around the distal ligand is not proline rich as observed for other cytochromes (Table 1 and Supplemental Figure 1). The measured redox potential of +155 mV for the MtoD heme is high for a bis-His coordinated heme and the heme group is significantly more solvent exposed than in monoheme cytochromes. It is possible that these unusual features of the MtoD heme group allow it to function as an electron donor to cytochrome bb_3_, cytochrome bc_1_ and/or CymA_ES−1_ (Figure 5).

Previous studies on the potentials of the heme groups of MtoA indicate that they span a mid-point potential range between +30 and −350 mV (Liu et al., 2012), which is lower than the potential of MtoD and indicates that electron transfer from MtoA to MtoD would be thermodynamically favorable. This supports the hypothesis that MtoD could be the electron transfer shuttle between the outer membrane MtoAB complex and the cytochrome bb_3_ oxidase on the cytoplasmic membrane, allowing the efficient generation of a PMF.

A membrane bound NADH dehydrogenase is proposed to reduce NAD^+^ to NADH using electrons provided by the quinol pool and energy generated by the PMF (Emerson et al., 2013). Either the cytochrome bc1 complex or CymA_ES−1_could generate quinol, using electrons supplied by MtoD (Figure 5). At +155 mV, the potential of MtoD is only slightly higher than the midpoint potential of ubiquinol (+100 mV vs. SHE) and consequently electron transfer from MtoD to cytochrome bc_1_ could occur in order to drive NADH production. The generation of reduced ubiquinol from cytochrome bc1 has been observed in other iron oxidizing bacteria, notably *A. ferrooxidans* (Elbehti et al., 2000). In this system, the two half reactions of the cytochrome bc1 complex run in reverse, causing the net transport of two protons across the cytoplasmic membrane for every ubiquinone reduced.

The role of CymA_ES−1_ is less clear. The homologous CymA cytochrome from *S. oneidensis* MR-1 was shown to be specific for menaquinone, that has a midpoint potential of −70 mV vs. SHE (McMillan et al., 2012). This suggests that the measured potential of MtoD would not allow thermodynamically favorable electron transfer to CymA_ES−1_. However, the highly exposed heme surface of MtoD is likely to be sensitive to changes in the local environment, such as those caused by protein complex formation. This may allow lowering of the MtoD midpoint potential on association with CymA_ES−1_, facilitating electron exchange and menaquinone reduction.

In summary, the structure and electrochemical properties of MtoD are consistent with its possible role as an electron shuttle between MtoAB on the outer membrane and a range of potential electron acceptors on the inner membrane. However, a full biochemical analysis will be required to verify the true redox partners and confirm the pathway of electron transfer through this iron oxidizing bacterium.

## Author contributions

CB, ME, ML, and LS carried out data acquisition and analysis. CB, ME, JB, DR, TC carried out data analysis and interpretation. CB, ME, ML, LS, JB, DR, TC drafted the manuscript. CB, ME, JB, DR, TC revised the manuscript. All authors approved the final manuscript.

### Conflict of interest statement

The authors declare that the research was conducted in the absence of any commercial or financial relationships that could be construed as a potential conflict of interest.
