# Supplementary material for: Characterization of MtoD from Sideroxydans lithotrophicus: a cytochrome c electron shuttle used in lithoautotrophic growth
Source: Front Microbiol. 2015 Apr 28;6:332. doi: 10.3389/fmicb.2015.00332 (PMC4412085; doi:10.3389/fmicb.2015.00332)
Supplement: Supplementary file 1 [file Table1.DOCX]

***Supplementary Material***

**Characterisation of MtoD from *Sideroxydans lithotrophicus:* a cytochrome c electron shuttle used in lithoautotrophic growth.**

Christopher R Beckwith^1^, Marcus J Edwards^1^, Matthew Lawes^1^, Liang Shi^2^, Julea N Butt^1^, David J Richardson^1^, Thomas A Clarke^1*^.

^1^Centre for Molecular and Structural Biochemistry, School of Biological Sciences and School of Chemistry, University of East Anglia, Norwich NR4 7TJ, United Kingdom

^2^Pacific Northwest National Laboratory, Richland, WA 99352, USA

**Correspondence:**

Dr Thomas Clarke, University of East Anglia, School of Biological Sciences, Norwich, NR4 7TJ, UK, Tom.clarke@uea.ac.uk

1. **Supplementary Figures and Tables**

## Supplementary Tables

**Supplementary Table 1** : **Crystallographic statistics for data reduction and refinement of MtoD crystals.**

|  |  |  |
| --- | --- | --- |
| **Data collection** | **SAD** | **Native** |
| Space group | P2_1_2_1_2 | P2_1_2_1_2 |
| Cell dimensions |  |  |
| a, b, c (Å) | 40.26, 92.29, 29.66 | 40.19, 92.17, 29.69 |
| A, β, ϒ (^o^) | 90, 90, 90 | 90, 90, 90 |
| Wavelength (Å) | 1.72 | 0.97 |
| Resolution (Å) | 2.29 | 1.47 |
| CC_1/2_ | 0.989 (0.529) | 0.998 (0.776) |
| Rpim (%) | 7.2 (40.6) | 3.7 (30.3) |
| I/σI | 14.29 (12.61) | 13.6 (2.5) |
| Completeness (%) | 96.1 (70.8) | 99.4 (98.8) |
| Redundancy | 10.8 (7.6) | 6.0 (5.5) |
| Refinement |  |  |
| Resolution (Å) |  | 1.47 |
| No. reflections used | | 18350 |
| Rwork/Rfree |  | 0.1943/0.2451 |
| No. atoms |  |  |
| Protein |  | 718 |
| Ligand |  | 43 |
| Water |  | 128 |
| B-factors |  |  |
| Protein |  | 17.556 |
| Ligand |  | 12.463 |
| Water |  | 30.509 |
| Bond lengths (Å) |  | 0.026 |

## Supplementary Figures

**Supplemental Figure 1: Sequence comparison of MtoD**. Alignment of the MtrC amino acid sequence against sequences of solved structures in the RCSB protein data bank revealed predominantly cytochromes C-551 and C-552 with sequence similarity. The conserved CXXCH motif is shown in bold, while the axial ligands to each heme iron are shown in red. Numbering for *S. lithotrophicus* includes signal peptide. Full organism names are (in descending order): *S. lithotrophicus* ES-1, *Nitrosomonas europaea*, *Pseudomonas aeruginosa*, *Hydrogenophilus thermoluteolus*, *Hydrogenobacter thermophilus*, *Pseudomonas stutzeri* and *Aquifex aeolicus*.
